# Atypical Presentation of Epstein-Barr Virus Infectious Mononucleosis With Cholestatic Hepatitis and Hyperbilirubinemia in a Young Adult: A Case Report

**DOI:** 10.7759/cureus.71066

**Published:** 2024-10-08

**Authors:** Chinmayi Pamala, Mohamed Orabi, Sachdev Avinash, Ibrahim Kamel

**Affiliations:** 1 Internal Medicine, Tufts University School of Medicine, Boston, USA; 2 Internal Medicine, Tufts Medical Center, Boston, USA; 3 Internal Medicine, Steward Carney Hospital, Dorchester, USA; 4 Internal Medicine, St. Elizabeth Medical Center, Boston University, Boston, USA

**Keywords:** case report, cholestatic hepatitis, epstein-barr virus, hyperbilirubinemia, infectious mononucleosis

## Abstract

This case report details the presentation of a 24-year-old male of South Asian descent with an atypical manifestation of Epstein-Barr virus (EBV) infectious mononucleosis, characterized by cholestatic hepatitis and hyperbilirubinemia. The patient initially presented with common symptoms of sore throat, intermittent fever, and general malaise, which subsequently progressed to include nausea and vomiting. Laboratory investigations revealed significantly elevated liver enzymes and bilirubin levels. Comprehensive serological testing confirmed an EBV infection. Despite the absence of typical risk factors, this case underscores the importance of considering EBV in the differential diagnosis for young adults presenting with both infectious symptoms and abnormal liver function tests. Early recognition of such atypical presentations is crucial for guiding appropriate management and avoiding unnecessary diagnostic procedures.

## Introduction

EBV, a prevalent human herpesvirus, is the primary cause of infectious mononucleosis, commonly known as *mono*. This illness typically presents with fever, sore throat, and lymphadenopathy, primarily affecting adolescents and young adults. Transmitted through saliva, EBV is often referred to as *the kissing disease* [1,2].

Most EBV infections are self-limiting and resolve without major complications. However, atypical presentations, such as cholestatic hepatitis and hyperbilirubinemia, can occur. Hepatic involvement is common in EBV infections, with 80%-90% of cases showing mild-to-moderate liver enzyme elevations. Cholestatic hepatitis, marked by significant jaundice and elevated bilirubin levels, is rare, occurring in less than 5% of cases [3-5].

This case report describes a 24-year-old male with an atypical presentation of EBV infectious mononucleosis, characterized by cholestatic hepatitis and hyperbilirubinemia. While EBV typically presents with fever, pharyngitis, cervical lymphadenopathy, fatigue, and splenomegaly, atypical manifestations such as hepatitis and jaundice can arise, especially in immunocompromised individuals. EBV is also linked to malignancies such as nasopharyngeal carcinoma, Burkitt lymphoma, Hodgkin’s lymphoma, and post-transplant lymphoproliferative disorder (PTLD), particularly in immunosuppressed individuals. Neurological complications, including meningoencephalitis, Guillain-Barré syndrome, and cranial nerve palsies, may also occur.

Gastrointestinal involvement, such as hepatitis or jaundice without classic mononucleosis symptoms, can complicate the diagnostic process by mimicking other hepatic or biliary conditions. This case underscores the need to consider EBV in the differential diagnosis of young adults presenting with infectious symptoms and abnormal liver function tests, even in the absence of typical risk factors like close contact with infected saliva, crowded living conditions, or immunosuppression [3,5].

## Case presentation

A previously healthy 24-year-old male presented to the emergency department with six days of infectious symptoms, including a sore throat, intermittent fevers, headache, and general malaise. He denied cough, rhinorrhea, or congestion and had taken only one 650 mg dose of acetaminophen in the past week. Three days before the presentation, he developed nausea and vomiting, accompanied by post-tussive epigastric pain. He denied abdominal pain, diarrhea, or constipation. Multiple home COVID-19 tests were negative. The patient had no history of intravenous drug use, recent travel, or exposure to sick contacts. He had received a new tattoo three months earlier but reported no rash or skin changes. He also noted a new sexual partner two months prior but denied any genital symptoms. Additionally, he had no known tick exposure.

On examination, the patient was afebrile (37.3 °C) but tachycardic with a heart rate of 106 bpm. His blood pressure was 137/91 mmHg, respiratory rate 18 breaths per minute, and oxygen saturation was 100% on room air. After fluid administration, his heart rate decreased to 88 bpm. The patient appeared in no acute distress, though scleral icterus was noted. His mucous membranes were moist, and tonsillar hypertrophy with exudates was observed. Mild cervical lymphadenopathy was palpated. Cardiovascular, respiratory, and abdominal exams were otherwise unremarkable.

Past Medical History

The patient had no previous medical conditions and was not on any medications. He reported rare alcohol consumption, and occasional marijuana use, and denied tobacco or illicit drug use. He had no history of sexually transmitted infections but reported recent sexual activity with a new partner.

Investigations

Bloodwork upon admission revealed notable findings, as shown in Table 1.

**Table 1:** Labs on admission. AST, aspartate aminotransferase; ALT, alanine aminotransferase

Test	Result	Reference range
Sodium	130 mEq/L	135-145 mEq/L
Potassium	3.4 mEq/L	3.5-5.0 mEq/L
Chloride	92 mEq/L	98-107 mEq/L
Calcium	8.5 mg/dL	8.5-10.5 mg/dL
Total bilirubin	5.4 mg/dL	0.1-1.2 mg/dL
Direct bilirubin	4.3 mg/dL	0.0-0.3 mg/dL
AST	567 U/L	10-40 U/L
ALT	601 U/L	7-56 U/L
Alkaline phosphatase	332 U/L	44-147 U/L
Albumin	3.6 g/dL	3.5-5.0 g/dL

Blood cell count was within normal limits, but the white blood cell differential showed lymphocytosis (6.1 x 10^9/L). Urinalysis was notable for moderate bilirubin. Hepatitis serologies were negative for Hepatitis A IgM antibody, Hepatitis B surface antigen, Hepatitis B core IgM antibody, and Hepatitis C antibody. Tests for syphilis, chlamydia, gonorrhea, and HIV were negative. An extensive polymerase chain reaction (PCR) viral respiratory panel was negative for influenza A and B, respiratory syncytial virus (RSV), human rhinovirus, human metapneumovirus, adenovirus, parainfluenza viruses (types 1-4), enterovirus, and SARS-CoV-2. The rapid strep test was negative. Gamma-glutamyl transferase (GGT) and prothrombin time/INR were normal.

**Figure 1:**
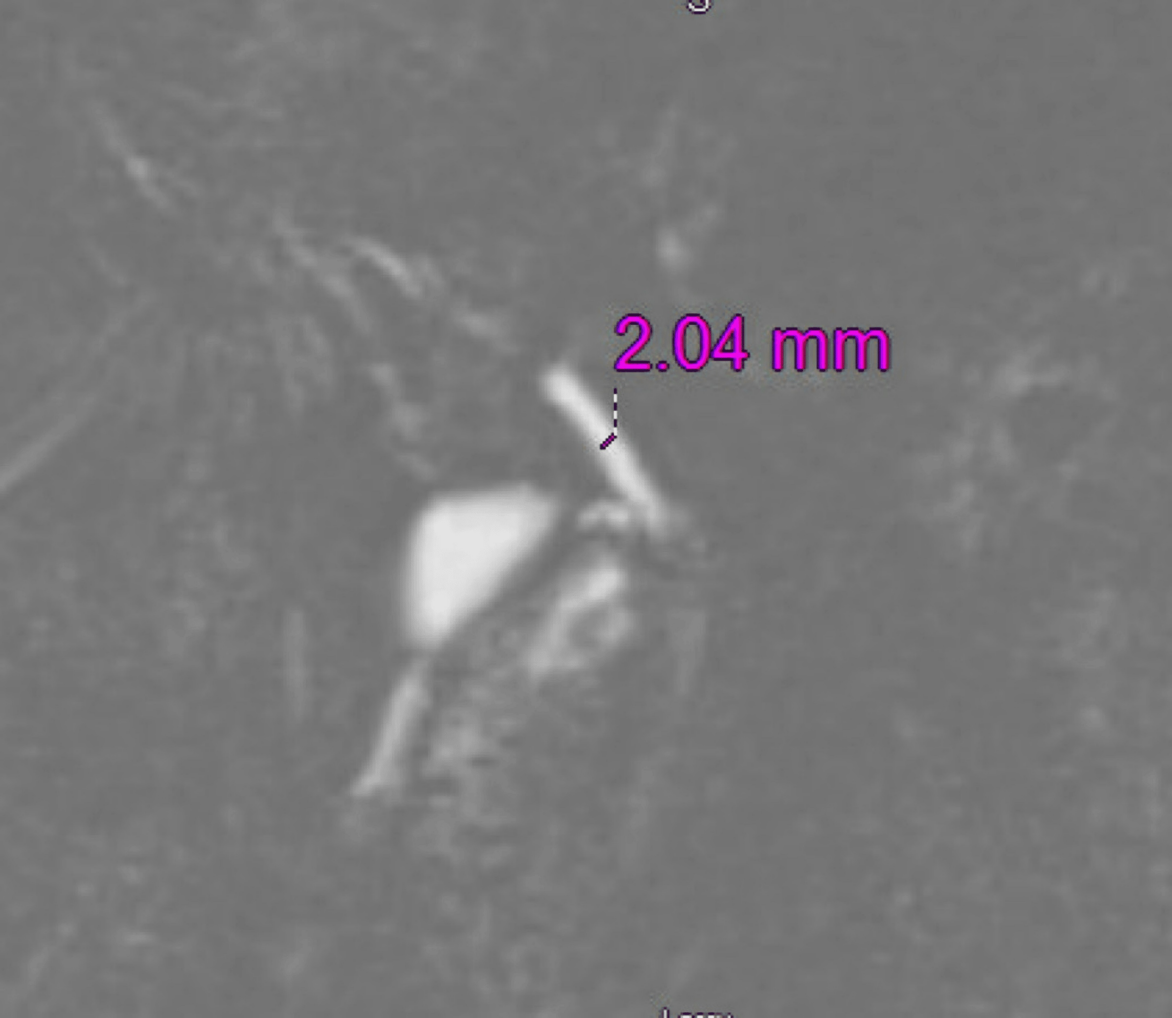
T2 3D MRCP showing biliary ducts: normal in caliber (CBD = 2 mm). MRCP, magnetic resonance cholangiopancreatography; CBD, common bile duct

An abdominal ultrasound showed no evidence of cholecystitis. Magnetic resonance cholangiopancreatography (MRCP) demonstrated a contracted gallbladder without gallstones, a normal common bile duct (Figure 1), and splenomegaly (15 cm). 

On day 4 of hospitalization, EBV serology returned positive, with an EBV capsid antigen IgM antibody >160 and EBV early antigen 48.8. The mono screen was also positive. Bloodwork on day 5 showed a peak bilirubin level of 6.9 mg/dL, with decreasing liver function tests (AST 298 U/L; ALT 629 U/L).

Differential Diagnosis

The differential diagnosis included a viral upper respiratory illness, with a high suspicion of EBV or influenza. Gastroenteritis was also considered given the vomiting and the patient's recent consumption of food from a new restaurant. Viral hepatitis was considered due to the recent tattoo and sexual activity. The rising AST/ALT and bilirubin levels raised concerns about choledocholithiasis or resolving cholangitis.

Management

The patient was managed conservatively due to the lack of evidence supporting the use of antivirals or steroids for EBV-related mononucleosis. IV fluids and IV ketorolac were administered for pain. Given his reduced PO intake from a sore throat, he received phenol spray and benzocaine lozenges. Ondansetron was given for nausea. On day 5 of hospitalization, the patient developed a fever of 102.9 °F, which resolved with acetaminophen. Due to the fever and a rising white blood cell count (from 8.5 to 10.4), blood cultures were obtained, and an ENT consultation was requested to evaluate the risk of superimposed bacterial pharyngitis.

## Discussion

EBV is a common infectious agent responsible for infectious mononucleosis, primarily affecting adolescents and young adults. The classic symptoms include fever, sore throat, fatigue, and lymphadenopathy. While most EBV infections are self-limiting, atypical presentations, such as cholestatic hepatitis and hyperbilirubinemia, are rare but significant. Cholestatic hepatitis is a rare complication of EBV infection, and jaundice is infrequently reported, particularly in younger individuals; it is more commonly observed in patients aged 35 and older [6-8]. When jaundice occurs in the context of EBV, it may be attributed to various causes, including autoimmune hemolytic anemia, cholestasis due to acalculous cholecystitis, biliary duct obstruction from abdominal lymphadenopathy, or direct cholestatic hepatitis [9,10]. The exact pathogenesis of cholestatic hepatitis associated with EBV remains unclear. It is unlikely that EBV directly infects hepatocytes, biliary epithelium, or vascular endothelium. Some theories suggest that cholestasis in EBV cases may be related to lipid peroxidation and the subsequent production of free radicals, contributing to liver injury [6,9].

Although mild elevations in liver enzymes are common, significant hyperbilirubinemia is unusual and can complicate the clinical picture, often prompting consideration of other etiologies, including viral hepatitis or biliary obstruction, before confirming EBV as the cause [5,11].

Current guidelines emphasize the importance of supportive care in managing EBV infections, as there is no specific antiviral treatment proven to reduce the severity or duration of the disease. Symptomatic management, including hydration, rest, and analgesics, remains the cornerstone of treatment. The use of corticosteroids is generally reserved for severe complications, such as airway obstruction or severe hemolytic anemia [5,12].

This case underscores the necessity of including EBV in the differential diagnosis for young adults presenting with both infectious symptoms and abnormal liver function tests, even in the absence of typical risk factors. Awareness of such atypical presentations can prevent unnecessary investigations and guide appropriate management strategies [5,12].

## Conclusions

This case highlights the importance of recognizing EBV as a potential cause of cholestatic hepatitis and hyperbilirubinemia, even in young adults without typical risk factors for liver disease. The unusual presentation of EBV in this patient emphasizes the need for clinicians to consider a broad differential diagnosis when encountering elevated liver enzymes and bilirubin levels in the context of infectious symptoms. Prompt identification of EBV can prevent unnecessary diagnostic investigations and support timely, appropriate management. This case contributes to the growing awareness of the diverse clinical manifestations of EBV and reinforces the value of a thorough and systematic approach in the evaluation of atypical cases.
